# Fracture risk after kidney transplantation: unchanged and unaddressed: a registry-based cohort study across two decades

**DOI:** 10.1093/ckj/sfag029

**Published:** 2026-02-06

**Authors:** Ina Karstoft Ystrøm, Christian Fynbo Christiansen, Per Ivarsen, Pieter Evenepoel, Ditte Hansen, Hanne Skou Jørgensen

**Affiliations:** Department of Clinical Medicine, Aarhus University, Aarhus, Denmark; Department of Renal Medicine, Aarhus University Hospital, Aarhus, Denmark; Department of Microbiology, Immunology and Transplantation, Nephrology and Renal Transplantation Research Group, Katholieke Universiteit Leuven, Leuven, Belgium; Department of Clinical Medicine, Aarhus University, Aarhus, Denmark; Department of Clinical Epidemiology, Aarhus University Hospital and Aarhus University, Aarhus, Denmark; Department of Renal Medicine, Aarhus University Hospital, Aarhus, Denmark; Department of Microbiology, Immunology and Transplantation, Nephrology and Renal Transplantation Research Group, Katholieke Universiteit Leuven, Leuven, Belgium; Department of Nephrology and Renal Transplantation, University Hospitals Leuven, Leuven, Belgium; Department of Nephrology, Copenhagen University Hospital – Herlev, Copenhagen, Denmark; Institute of Clinical Medicine, University of Copenhagen, Copenhagen, Denmark; Department of Clinical Medicine, Aarhus University, Aarhus, Denmark; Department of Renal Medicine, Aarhus University Hospital, Aarhus, Denmark; Department of Clinical Medicine, Aalborg University, Aalborg, Denmark

**Keywords:** chronic kidney disease-mineral and bone disorder, bone fracture, epidemiology, kidney transplantation, osteoporosis

## Abstract

**Background:**

Kidney transplant recipients face a high risk of adverse events, including fractures. Reported fracture rates post-transplant vary widely and may have changed over time. The consequences of a fracture, especially subsequent fractures, remain under-investigated. This study examined the risk and prognosis of fractures after kidney transplantation in the current era.

**Methods:**

This retrospective cohort study included all adults who received a first single-organ kidney transplant between 2000 and 2022 in Denmark. Nationwide healthcare registries were utilized to identify the study population and provide demographic data, diagnostic and procedural codes, and prescription data. Cumulative incidence (risk) of both first fracture and subsequent fracture was computed, treating death as a competing risk. Crude, and sex- and age-standardized incidence rates were estimated for different time periods.

**Results:**

The study included 3977 first kidney transplant recipients, of whom 788 sustained any post-transplant fracture. The 10-year risk of fracture was 21% (95% confidence interval 20–23). Crude incidence rates of any fracture remained unchanged over time, while standardized estimates showed a slight decline. Fractures were primarily located at the peripheral skeleton. Twenty-eight percent of patients with a fracture sustained a subsequent fracture, with the highest incidence at 6–12 months following the first event.

**Conclusions:**

Kidney transplant recipients remain at high fracture risk, with unchanged fracture rates over the past 20 years. The imminent fracture risk following a first fracture is high and largely unaddressed. Although formal guidelines have yet to be established, this knowledge should urge clinicians to perform risk assessment and consider intervention following a post-transplant fracture.

## Graphical Abstract

**Graphical Abstract:**
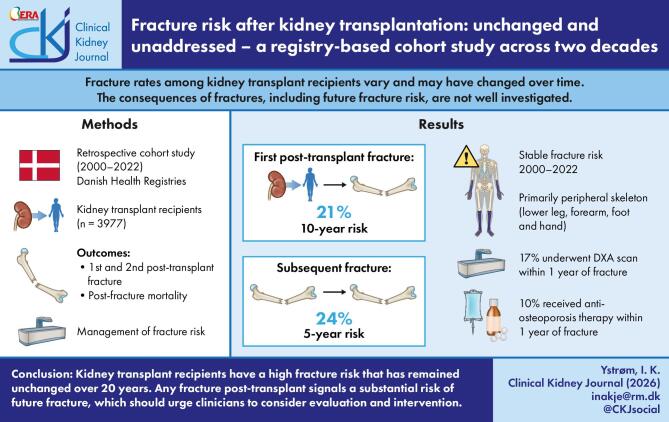

KEY LEARNING POINTS **What was known:**

- Reported fracture rates among kidney transplant recipients vary widely and may have changed with general treatment improvements in recent decades.
- The consequences of a fracture, including mortality and risk of subsequent fractures, remain under-investigated.

**This study adds:**

- One in five kidney transplant recipients will experience any fracture within 10 years post-transplant.
- Fracture risk post-transplant is largely unchanged over the past two decades.
- Kidney transplant recipients who fracture are at increased risk of subsequent fractures, including those with minor, peripheral skeletal fractures.

**Potential impact:**

- Our results emphasize the continued disease burden caused by bone fragility and the overlooked risk of subsequent fracture following a first post-transplant fracture.
- These findings highlight the importance of risk evaluation after a first fracture, irrespective of anatomical location, as well as the imperative for appropriate clinical guidelines.

## INTRODUCTION

Kidney transplantation offers superior outcomes for individuals with kidney failure compared with other replacement therapies [1, 2]. Advances in immunosuppressive therapy have improved graft survival and extended recipient life expectancy over the last decades [3]. However, kidney transplant recipients remain at high risk of adverse outcomes, including fractures, compared with the background population [4].

The reported post-transplant fracture risk varies widely, with a 5-year cumulative incidence ranging from 1% to 27% [5]. This variability may reflect differences in fracture types, data ascertainment methods and era. In kidney transplant recipients, fracture risk relates to both traditional osteoporotic risk factors and transplant-specific factors, including pre-existing damage to bone from kidney failure, immunosuppressive therapy and disturbances of mineral metabolism [6], all of which may have changed during the last decades. The focus on glucocorticoid-sparing protocols in recent decades may have reduced fracture risk [7–9]. However, data on temporal trends in post-transplant fracture risk are limited [10, 11]. Further, many studies focused on major osteoporotic fractures (MOF) [12] and may have overlooked the clinical relevance of peripheral fractures.

Recent evidence points towards an increased mortality rate in patients following a fracture [12, 13], while the risk of a subsequent fracture after an initial event is largely unknown. If a first fracture reflects underlying skeletal fragility, it may present a critical opportunity for clinical intervention.

This study investigates fracture risk among transplant recipients within the context of contemporary glucocorticoid-sparing protocols. Key outcomes include temporal trends, clinical consequences and management of fractures post-transplant.

## MATERIALS AND METHODS

### Study design and setting

This was a retrospective cohort study utilizing national health registries in Denmark. The Danish Nephrology Registry (DNR) was used to identify the study population and provide demographic data [14]. This registry contains information on all patients who have undergone kidney replacement therapy (dialysis or transplantation) in Denmark. The Danish National Patient Registry, DNPR [15], was used to obtain further information on patient characteristics and outcome data. The DNPR has collected diagnoses from the public healthcare system in Denmark since 1978. The unique civil registration number [16] was used to link between registries.

### Study population

**Figure 1:**
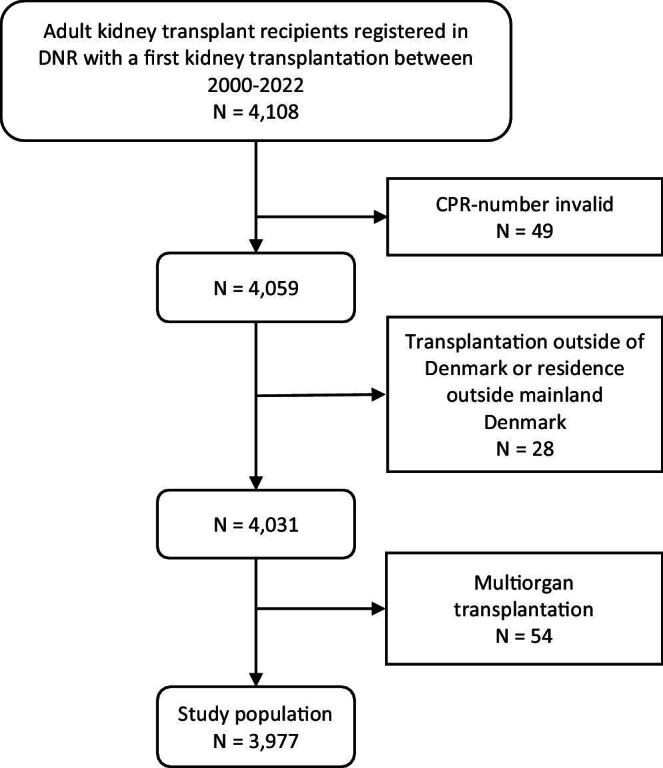
Flow chart of kidney transplant recipients.

The study population consisted of adult patients who received a first kidney transplantation between 1 January 2000 and 31 December 2022 as registered in the DNR. Exclusion criteria were invalid civil registration number, transplantation performed outside of Denmark, patients living outside mainland Denmark (the Faroe Islands or Greenland) and multiorgan transplantation (Fig. 1).

### Transplantation center and immunosuppression protocols

The standard immunosuppressive treatment protocols for the four centers performing kidney transplantation in Denmark during the study period are given in Supplementary data, Table S1. Briefly, standard triple immunosuppressive therapy with oral glucocorticoids, a calcineurin inhibitor (ciclosporin or tacrolimus) and an antimetabolite (azathioprine or mycophenolate) was initiated at the time of transplantation [17]. Induction and maintenance glucocorticoids were standard of care at all centers except one (Odense), where a steroid-free protocol following induction was in effect for the entire study period [18]. Methylprednisolone was the first-line therapy for acute kidney graft rejections at all centers. Information on immunosuppressive therapy including corticosteroids on an individual level was not available.

### Ascertainment of events

Classification codes by the Danish modification of the International Classification of Disease 8th (ICD-8, before 1994) or 10th (ICD-10) edition, as registered in the DNPR, were used to identify fractures (Supplemental Codebook 1). In Denmark, fractures are managed within the tax-based Danish hospital system, and the capture of fracture events in the DNPR is highly reliable [19]. Both primary and secondary diagnoses were included for all inpatient, outpatient and emergency hospital contacts. Fractures were grouped into categories based on anatomical sites consisting of: thoracolumbar spine; hip; proximal humerus; forearm (together defining MOF); cervical spine, ribs, and sternum; shoulder and clavicula; hand; other arm; pelvis; lower leg and knee; foot; other femur (not hip); and multiple fracture locations. Fractures of the skull, face, fingers and toes were not included. Knowledge on trauma type was not available. Incident fractures were the first fracture occurring after the date of transplantation. Subsequent fractures were the next fracture occurring after a fracture-free window of 30 days (different anatomical location) or 90 days (same location) [20, 21] (Codebook 2). Death-censored graft failure, defined as initiation or return to dialysis or re-transplantation, was obtained from the DNR. Information on death or emigration was derived from the Danish Civil Registration System.

### Covariates

Information on demographics, primary kidney disease, dialysis therapy, transplantation center, kidney donor type and acute rejections was obtained from the DNR. The primary kidney disease was defined by the ERA Registry Primary Renal Disease codes (Codebook 3) [22]. Comorbidities were obtained from the DNPR as registered diagnoses up to 10 years before the date of transplantation. Dual-energy X-ray absorptiometry (DXA) scans were identified by radiology-specific procedure codes (DNPR). Information on anti-osteoporotic therapy (bisphosphonates, denosumab, romosozumab, strontium ranelate and teriparatide) was ascertained from redeemed prescriptions as registered in the Danish National Prescription Registry (Lægemiddelstatistikregisteret, or LSR) [23] and as in-hospital procedure codes [bisphosphonates and denosumab (DNPR)]. Registrations were captured from 1 year before to 1 year after the transplantation date, and from the date of first post-transplant fracture to 1 year later (Codebooks 3 and 4). Treatment with phosphate binders, active vitamin D and calcimimetics was provided to the patients directly from the treatment center, and thus, this information was not available through the LSR.

### Statistical analyses

Continuous variables are presented as median with interquartile range (IQR), and categorical variables are presented as counts with percentages. Patients were followed until the date of first post-transplant fracture, emigration, death or end of the study period (31 December 2022). The 5- and 10-year cumulative incidence (risk) of any post-transplant fracture and MOF was computed, treating death as a competing risk. The incidence rate of a first fracture was calculated as number of first fractures by 1000 person-years. Incidence rates by post-transplant 6-month intervals were calculated as number of events by person-risk time during that interval. Incidence rates by calendar period of transplantation (2000–04, 2005–09, 2010–14, 2015–19, 2020–22) were computed as crude and direct standardized to the age- and sex-distribution of patients transplanted in the first period (2000–04). For this, follow-up was limited to the first five post-transplant years. Rates were compared, assessing the rate difference with 95% confidence interval (CI). The proportion of patients who underwent DXA scan and/or received anti-osteoporosis therapy was reported.

The risk of fracture depending on at least one rejection episode within the first post-transplant year, and the risk of fracture depending on transplant center were investigated by competing risk analysis. For this analysis, transplantations at Odense (steroid-free protocol) were compared with Aarhus and Rigshospitalet (contemporary steroid-containing protocols). To assess factors associated with fracture, we fitted a Cox proportional hazards model with shared frailty. The shared frailty was specified to account for unmeasured center-level heterogeneity. Covariates modeled as fixed-effects included standard treatment protocol (corticosteroid-free versus corticosteroid-containing), sex, age, diabetes, pre-transplant dialysis vintage, history of fracture, donor type and time interval of transplantation [24]. The association between incident fracture and mortality was evaluated in a standard Cox model treating fracture and graft loss as time-varying covariates and adjusted for age, sex, diabetes, cardiovascular disease and donor type. Lastly, the association between incident fracture and graft loss treating fracture as a time-varying covariate and excluding patients with graft loss before fracture event was adjusted for age, sex, primary kidney disease and donor type. For all models, assumptions of proportional hazards were checked using log(-log) plots and found appropriate.

In a sensitivity analysis, kidney graft loss was treated as a competing event, and the incidence rate and cumulative incidence of a first fracture were estimated for patients with a functioning graft only.

For subanalyses, patients with incident fractures were followed until emigration, death or end of study. The 1- and 2-year mortality risk following any first fracture and grouped fracture locations were computed.

The risk of a subsequent fracture was computed as the cumulative 2- and 5-year risks, treating death as competing event and incidence rates. Imminent fracture risk was assessed using 6-month interval incidence rates following the initial fracture. Analyses and illustrations were performed using R version 4.2.2 (R Foundation for Statistical Computing, Vienna, Austria). The study complies with the STrengthening the Reporting of OBservational studies in Epidemiology (STROBE) statement [25].

## RESULTS

### Baseline characteristics

The study population consisted of 3977 first-time recipients of a single-organ kidney transplantation (Fig. 1). Demographic data are given in Table 1. Median age at transplantation was 50 years, 37% were female, 23% had diabetes mellitus and 13% had a pre-transplant fracture. A DXA scan was registered for 6% of patients in the year before kidney transplantation and 11% of patients in the first post-transplant year. The proportion of patients with a DXA scan increased in recent years (2021: 28%). Anti-osteoporotic therapy was noted in 1.6% of patients in the first post-transplant year.

**Table 1:** Baseline characteristics of Danish kidney transplant recipients, 2000–22.

	*N* = 3977^a^
Age, years	50 (40, 60)
Sex, female, *n* (%)	1487 (37)
Calendar year of transplantation, *n* (%)	
2000–10	1577 (40)
2011–22	2400 (60)
Transplant center, *n* (%)	
Aarhus	1434 (36)
Copenhagen	
Herlev^b^	193 (5)
Rigshospitalet	1344 (40)
Odense	1006 (25)
Primary kidney disease, *n* (%)	
Diabetes mellitus	586 (15)
Familial/hereditary nephropathies	695 (18)
Glomerular disease	1164 (29)
Other systemic disease	34 (1)
Renal vascular disease/hypertension	400 (10)
Tubulointerstitial disease	334 (8)
Unspecified renal disorder	764 (19)
Pre-transplant dialysis therapy, *n* (%)	
Hemodialysis	1957 (49)
Peritoneal dialysis	1227 (31)
Pre-emptive	793 (20)
Time on dialysis, months	22 (11, 42)
Donor type, *n* (%)	
Deceased donor	2591 (65)
Living donor	1386 (35)
Comorbidity diagnoses up to 10 years	
before transplantation date, *n* (%)	
Hypertension	2809 (71)
Cardiovascular disease	1405 (35)
Diabetes	901 (23)
Any fracture	503 (13)
Major osteoporotic fracture	176 (4)
Time from last fracture to transplantation, years	4.0 (1.8, 6.7)
Charlson comorbidity index	2.0 (2.0, 4.0)
History of parathyroidectomy, *n* (%)	222 (6)

a Continous variables are presented as median (IQR). Categorical variables are presented as counts (%).

^b^Transplant center for the period 2000–10. Major osteoporotic fracture: thoracolumbar spine, hip, proximal humerus, forearm.

### Fracture risk

**Figure 2:**
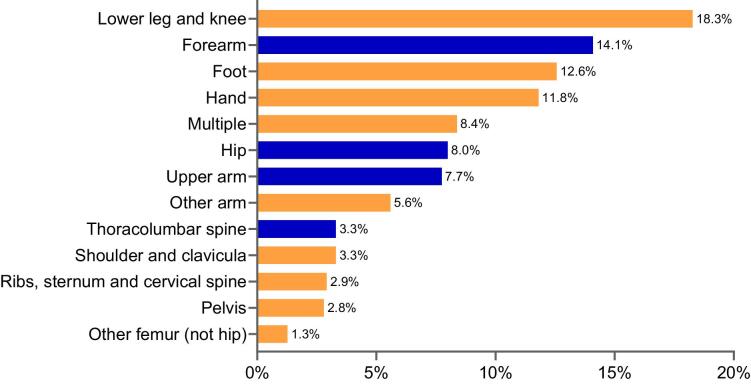
Location of first post-transplant fracture (%). MOF are depicted in dark blue.

The median follow-up was 6.2 years (IQR 2.8–11.1). A total of 757 patients (19%) died and 788 (20%) sustained any first post-transplant fracture. Most fractures were located at the peripheral skeleton, with fractures of the lower leg, forearm, foot and hand comprising 57% of first post-transplant fractures (Fig. 2). Extending the follow-up until a first MOF yielded 363 MOF events (9%). Five- and 10-year risks and incidence rates, of any fracture and MOF are given in Table 2 and Supplementary data, Fig. S1. In patients with fracture, the median time from transplantation to fracture was 4.2 years. The risk of fracture was highest between 1 and 2.5 years (Supplementary data, Fig. S2).

**Table 2:** Cumulative incidence and incidence rate of any first post-transplant fracture and first post-transplant MOF.

	5-year cumulative incidence, % (95% CI)	10-year cumulative incidence, % (95% CI)	Incidence rate per 1000 person-years (95% CI)
Any fracture (*n* = 3997)	12.9 % (95% CI 11.8–14.0)	21.2% (95% CI 19.8–22.8)	26.8 (95% CI 25.0–28.8).
MOF (*n* = 3997)	5.1 % (95% CI 4.4–5.8)	9.2% (95% CI 8.2–10.3)	11.3 (95% CI 10.2–12.6).

MOF includes fractures of thoracolumbar spine, hip, proximal humerus or forearm.

### Temporal changes in fracture risk

Crude fracture incidence was unchanged across intervals of calendar time in the study period. Recipient age rose from median 46 years (IQR 36–55) in 2000–04 to 54 years (43–63) in 2020–22, while pre-emptive transplants increased and pre-transplant dialysis time decreased.

**Figure 3:**
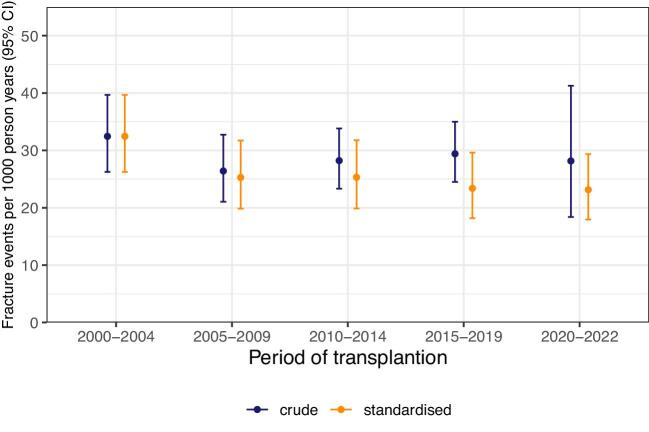
Crude and standardized incidence rates for any first post-transplant fracture by calendar period of kidney transplantation. Estimates restricted to 5 years of follow-up.

After age and sex standardization, there was a marginal decrease in fracture incidence (Fig. 3). This was also the case for MOF (Supplementary data, Fig. S3).

### Factors associated with fracture

Patients who sustained a first fracture were more likely to be women, and to have diabetes, a history of fracture and a longer time on dialysis leading up to transplantation compared with patients who did not fracture (Supplementary data, Table S2). The 10-year risk of fracture was marginally higher in patients with rejections than in patients without [22.8% (95% CI 19.2–26.6) versus 21.0% (95% CI 19.4–22.6)] (Supplementary data, Fig. S4). The unadjusted 10-year risk of fracture was lower in patients transplanted at Odense (steroid-free immunosuppressive standard protocol) compared with Aarhus and Rigshospitalet [18.7% (95% CI 15.9–21.6) versus 22.3% (95% CI 20.5–24.2)] (Supplementary data, Table S3 and Fig. S5). Differences in baseline characteristics are presented in Supplementary data, Table S4. In the Cox model with shared frailty, transplantation under a corticosteroid-free standard protocol was associated with a lower fracture risk compared with transplantation under a corticosteroid-containing protocol after adjusting for age, sex, diabetes, pre-transplant dialysis vintage, history of fracture, donor type and time period of transplantation [adjusted hazard ratio (aHR) 0.79, 95% CI 0.67–0.94] (Table 3). When the same model was fitted without shared frailty, the effect estimates remained nearly identical.

**Table 3:** Cox model with shared frailty modeling factors associated with any fracture.

Characteristic	aHR	95% CI	*P*
Standard treatment protocol			
Corticosteroid-containing^a^	Ref		
Corticosteroid-free^b^	0.79	0.67, 0.94	.018
Age, years	1.03	1.02, 1.03	<.001
Sex, female	1.33	1.15, 1.53	<.001
History of diabetes, yes	1.66	1.42, 1.93	<.001
Dialysis treatment			
Pre-emptive	Ref		
Short dialysis duration (≤18 months)	1.03	0.82, 1.28	.83
Long dialysis duration (>18 months)	1.27	1.03, 1.56	.03
History of any fracture			
No	Ref		
Yes (≤1 year before tx)	1.91	1.22, 2.99	.005
Yes (>1 year before tx)	1.77	1.47, 2.15	<.001
Living donor	0.90	0.76, 1.06	.21
Calendar year of transplantation			
2000–04	Ref		
2005–09	0.91	0.74, 1.11	.35
2010–14	0.94	0.77, 1.16	.58
2015–19	0.95	0.75, 1.20	.65
2020–22	0.81	0.53, 1.26	.35

^a^Includes centers: Aarhus, Herlev, Rigshospitalet.

^b^Includes center: Odense. tx, transplantation.

### Post-fracture outcomes

**Figure 4:**
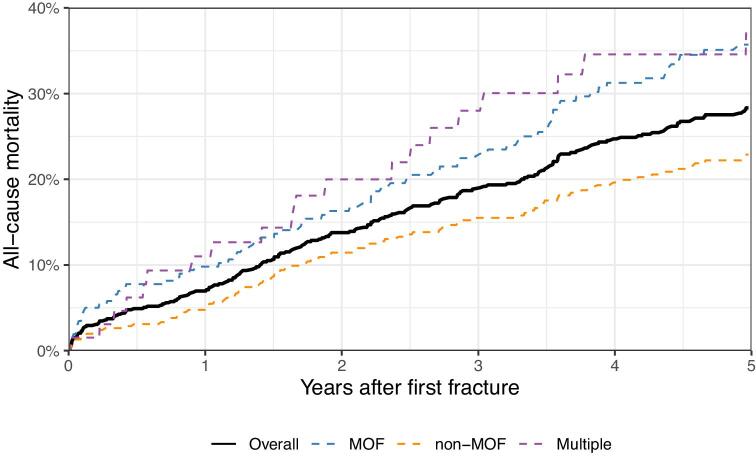
The mortality risk after first post-transplant fracture, overall and by first fracture category. Non-MOF, any other fracture not classified as MOF; multiple, fracture episodes with multiple fracture locations.

Mortality curves following a fracture are shown in Fig. 4 [5-year mortality 28.3% (95% CI 24.9–31.9)]. Fracture patients had a higher risk than non-fracture patients (Table 4a), with hip fracture showing particularly elevated mortality (aHR 2.69, 95% CI 2.04–3.56). After a hip fracture, the 30-day mortality was 11.3% (95% CI 3.0–18.8). There was no increased risk of death-censored graft failure in patients with fractures versus patients without fractures (Table 4b).

**Table 4a:** aHRs for the risk of all-cause mortality by fracture status (any fracture and hip fracture).

	Univariate HR (95% CI)	*P*	Age + sex aHR (95% CI)	*P*	Multivar. aHR^a^(95% CI)	*P*
Mortality (*n* = 3977)						
Model for any fracture						
Any first fracture (*n* = 788)	1.71 (1.50–1.96)	<.001	1.40 (1.23–1.61)	<.001	1.29 (1.14–1.46)	<.001
No fracture (*n* = 3189)	Ref		Ref			
Model for hip fracture						
First hip fracture (*n* = 73)	4.64 (3.51–6.12)	<.001	3.17 (2.4–4.2)	<0.001	2.69 (2.04–3.56)	<.001
No first hip fracture (*n* = 3904)	Ref		Ref		Ref	

^a^Multivariate estimate adjusted for age, sex, diabetes, cardiovascular disease, donor type, graft loss (as time-varying).

**Table 4b:** aHRs for the risk of death-censored graft-failure by fracture status (any fracture).

	Univariate HR (95% CI)	*P*	Age + sex aHR (95% CI)	*P*	Multivar. aHR^a^(95% CI)	*P*
Death-censored graft failure (*n* = 3857)						
Any first fracture (*n* = 668)	0.95 (0.76–1.20)	.68	1.01 (0.80–1.11)	.9	0.99 (0.78–1.25)	>.9
No fracture (*n* = 3189)	ref		ref		Ref	

^a^Multivariate estimate adjusted for age, sex, primary kidney disease, donor type.

### Imminent fracture risk

**Figure 5:**
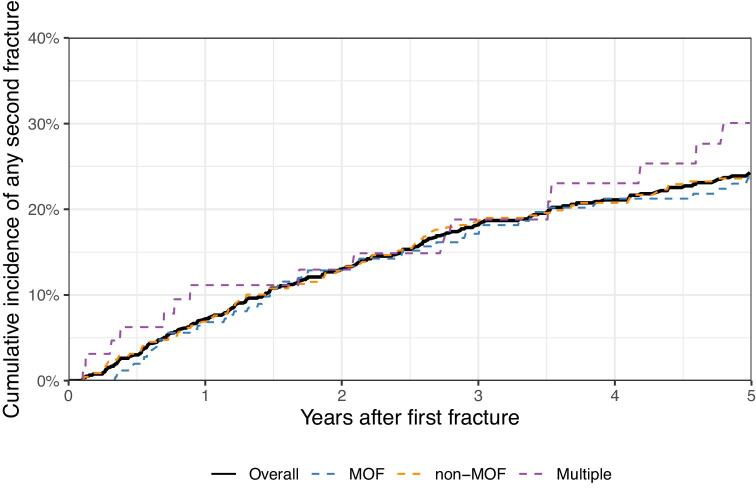
Risk of subsequent fracture, overall and by first fracture category. Non-MOF, any other fracture not classified as MOF; multiple, fracture episodes with multiple fracture locations.

At least one subsequent fracture occurred in 220 patients (28%) following a first post-transplant fracture. The median time from the first to the second fracture was 2.5 years (IQR 1.0–5.2). MOF and non-MOF events carried a similar risk of sustaining any second fracture (5-year risk of 24.3% vs 23.6%) (Fig. 5), with a first fracture of the hip, pelvis and spine carrying the highest 2-year cumulative incidence (Supplementary data, Table S5). Minor skeletal fractures resulted in a similar risk of the next fracture being a MOF as was seen after a first MOF event (5-year risk 7.8% versus 9.9%) (Table 5). The highest crude incidence of subsequent fracture was 93 per 1000 person-years (95% CI 63–133) at 6–12 months after the first fracture (Supplementary data, Fig. S6).

**Table 5:** Risk of any fracture, MOF and non-MOF by first fracture type.

	Patients at risk	2-year cumulative incidence, % (95% CI)	5-year cumulative incidence, % (95% CI)	Incidence rate per 1000 person-years (95% CI)
Risk any fracture by first fracture type				
Overall	788	13 (11–16)	24 (21–28)	64 (56–73)
Previous MOF	261	13 (9.0–18)	24 (19–30)	72 (57–90)
Previous non-MOF	461	13 (10–17)	24 (19–28)	58 (48–69)
Previous multiple fractures	66	13 (6.0–23)	30 (18–43)	81 (49–125)
Risk of MOF by first fracture type				
Overall	788	4.8 (3.4–6.6)	8.5 (6.6–11)	21 (16–25)
Previous MOF	261	6.2 (3.6–9.7)	10 (6.4–14.4)	29 (20–41)
Previous non-MOF	461	4.1 (2.5- 6.3)	7.8 (5.4–11)	17 (12–22)
Previous multiple fractures	66	4.9 (1.3–12)	9.6 (3.3–20)	19 (7.0–42)
Risk of non-MOF by first fracture type				
Overall	788	5.5 (4.0–7.4)	12 (9.2–14)	29 (24–35)
Previous MOF	261	5.0 (2.7–8.3)	12 (8.3–17)	28 (20–40)
Previous non-MOF	461	7.3 (5.1–10)	14 (10–17)	29 (23–37)
Previous multiple fractures	66	5.0 (1.3–13)	16 (7.2–17)	35 (17–64)

Among all patients who sustained a fracture post-transplant, 17% subsequently underwent a DXA scan, and 10% received anti-osteoporotic therapy (bisphosphonates, denosumab, romosozumab, strontium ranelate or teriparatide) within 1 year of the fracture event. These proportions increased slightly for patients, who fractured in the recent years (2021: 25% DXA scan, 17% therapy).

### Sensitivity analyses

We performed a sensitivity analysis, in which both kidney graft loss and death were treated as competing events. This yielded risk estimates similar to the main analysis (Supplementary data, Table S6).

## DISCUSSION

This nationwide, registry-based study demonstrates that fracture risk remains high in contemporary kidney transplant recipients, with one in five patients experiencing a fracture in the 10 years following kidney transplantation. Moreover, fracture risk was largely unchanged. Patients who fractured were at increased risk of subsequent fractures and death, and this was also true for minor fractures, which were particularly common in the post-transplant period.

The fracture risk found in this study is substantially higher than expected for a matched background population [4]. Fracture risk was highest in the first 5 years post-transplant, but we found no evidence of a particularly elevated risk in the first year, contrary to previous reports [26]. Established risk factors such as older age, diabetes and long time on dialysis [24] were also evident in this study. In addition, the fracture risk was lower for patients transplanted under a corticosteroid-free standard protocol compared with those transplanted at centers with steroid-minimization. This result should be interpreted cautiously. First, individual-level information on steroid exposure was not available and therefore could not be accounted for. Second, although a shared frailty model was employed to address clustering by center, the small number of centers limits the model’s ability to capture true between-center variability. Consequently, unmeasured center-level practice differences (including follow-up strategies and fall- or fracture-prevention practices), as well as residual patient-level differences, may remain. However, findings align with reports from the USA, showing a decreased fracture risk in kidney transplant patients discharged without versus with corticosteroid therapy [27].

Fracture risk remained stable over two decades. Multiple, potentially opposing factors may influence this persistent risk. In recent years, shorter pre-transplant time spent in kidney failure may ameliorate pre-existing damage to bone, while increased patient age and a general expansion of eligibility criteria as seen across Europe [28] may countervail expected long-term gains. However, in our analyses, even age- and sex-adjusted estimates of fracture risk only marginally declined over time. These findings underline the ongoing challenge of maintaining bone health after kidney transplantation despite general improvements in therapy.

The occurrence of a fracture post-transplant was associated with an increased risk of subsequent fracture as well as mortality. While the mortality risk was highest after hip and vertebral fractures, the less studied, minor fractures carried a similar risk of subsequent fractures, including a later MOF. Fractures at the peripheral skeleton, including lower leg, forearm, foot and hand, were the most common fracture type post-transplant. Relative to the general population, our results indicate that fractures of the lower leg and foot are more frequent in kidney transplant recipients, whereas the risk of hip fracture is lower compared with both the general population and patients on dialysis [4, 29, 30]. This aligns with recent findings indicating an overall stability of bone density at the central skeleton post-transplant, but evidence of ongoing bone loss at peripheral skeletal sites [9, 31, 32]. With steroid minimization, sustained hyperparathyroidism may elevate fracture risk at sites rich in cortical bone, such as the long bones, metacarpals and metatarsals [9, 33–35]. These fractures may be overlooked in clinical practice and in research; however, as was demonstrated in this study, peripheral fractures indicate bone fragility to a similar degree as MOF, judged by the similar risk of future fracture.

The risk of a subsequent fracture was almost triple in the first year following the first fracture, supporting the concept of “imminent fracture risk” [36, 37]. This finding highlights the need for timely intervention following a fracture event. Clinical practice guidelines, however, remain vague on how to address fracture risk in the post-transplant period [7, 38]. Yet, while treatment options for patients with estimated glomerular filtration rate (eGFR) <30 mL/min/1.73 m^3^ remain unestablished, a recent European consensus paper recommended that patients with eGFR >30 mL/min/1.73 m^3^ be eligible to standard anti-osteoporotic therapy [39]. We show that under current practice, only a subset of patients receives anti-osteoporosis therapy within a year of fracture. The same finding applies to the use of DXA scans, which are reasonable to perform in cases of suspected bone fragility [40]. Accordingly, future treatment strategies could very likely be optimized to the benefit of patients.

Strengths of this study include the large cohort size and completeness of data facilitated by the utilization of nationwide health registries. Of note, all fractures are managed within the tax-based Danish hospital system, with no clinics outside of this system treating fractures. Hence, the capture of fracture events in the Danish healthcare registries is close to complete [19]. Limitations include the retrospective design and the lack of individual-level data on chronic kidney disease–mineral and bone disorder targeted therapy, immunosuppressive therapy including corticosteroids, bone density results, fracture risk profile (height, weight, diet, physical function, family history, tobacco and alcohol use) or fracture trauma mechanism. Since many vertebral fractures are asymptomatic [41] these are likely under-reported in this study. Collapsing fracture registrations into fracture episodes within time windows was considered the most precise strategy for handling serial contacts, but it cannot rule out that independent registrations were incorrectly combined or that dependent registrations were mistakenly counted as new fracture episodes. Results may not generalize to other ethnic groups, as the study population was predominantly Caucasian and from a background population with a relatively high fracture incidence [42].

In conclusion, this study demonstrates that fracture risk remains a major challenge in kidney transplant recipients even under steroid minimization protocols. We emphasize the continued disease burden caused by bone fragility and the overlooked risk of subsequent fracture following a first event. Future studies on fracture risk assessment and treatment should be prioritized in this patient population.

## Supplementary Material

sfag029_Supplemental_File

## Data Availability

The research data underlying this study cannot be shared according to Danish law.
