# Supplementary material for: Fracture risk after kidney transplantation: unchanged and unaddressed: a registry-based cohort study across two decades
Source: Clin Kidney J. 2026 Feb 6;19(3):sfag029. doi: 10.1093/ckj/sfag029 (PMC13098143; doi:10.1093/ckj/sfag029)
Supplement: sfag029_Supplemental_File [file sfag029_Supplemental_File.docx]

| **Supplemental content:**  **Codebook, Tables, Figures.**  **Fracture risk after kidney transplantation: Unchanged and unaddressed – a registry-based cohort study across two decades** |
| --- |

**Table of contents:**

1. Codebooks

2. Supplemental tables

3. Supplemental figures

4. References

## 1 Codebooks

**Codebook 1 – Hospital diagnoses of fracture**

| **Variable/disorder** | **Subgroups** | **Type of code** | **Code list** | **Data source** |
| --- | --- | --- | --- | --- |
| Fracture, all | Cervical spine | ICD-10 | DS120, DS121*, DS122*, DS127, DS129, | DNPR |
|  |  | ICD-8 | 80500-80510 | DNPR |
|  | Ribs and sternum | ICD-10 | DS222-DS229 | DNPR |
|  |  | ICD-8 | 80700-80793 | DNPR |
|  | Pelvis | ICD-10 | DS321-DS325, DS327B, DS328C, | DNPR |
|  |  | ICD-8 | 80800-80899 | DNPR |
|  | Shoulder and clavicula | ICD-10 | DS420*-DS421,  DS427*-DS429, | DNPR |
|  |  | ICD-8 | 81009-81199 | DNPR |
|  | Other arm | ICD-10 | DS424*  DS520*-DS524*,  DS527-DS529* | DNPR |
|  |  | ICD-8 | 81220-81229, 81299,  81300-81319 | DNPR |
|  | Hand | ICD-10 | DS620-DS628*  Excluding phalanges DS625*, DS626*, DS627*. | DNPR |
|  |  | ICD-8 | 81400–81599 | DNPR |
|  | Femoral, not hip | ICD-10 | DS723-DS729* | DNPR |
|  |  | ICD-8 | 82100-82199 | DNPR |
|  | Lower leg and knee | ICD-10 | DS820-DS829* | DNPR |
|  |  | ICD-8 | 82200-82399,  82400-82499 | DNPR |
|  | Foot | ICD-10 | DS920-DS929*  Excluding phalanges DS924, DS925* | DNPR |
|  |  | ICD-8 | 82500–82599 | DNPR |
| Unspecified | Multiple | ICD-10 | DT02* | DNPR |
|  |  | ICD-8 | 82709-82799 | DNPR |
|  | Upper extremity | ICD-10 | DT109 | DNPR |
|  | Lower extremity | ICD-10 | DT129 | DNPR |
| Fracture (Major Osteoporotic Fractures, MOF) | Hip | ICD-10 | DS720, DS721*, DS722 | DNPR |
|  |  | ICD-8 | 82000-82019 | DNPR |
|  | Thoracolumbar spine | ICD-10 | DS220*, DS221,  DS320*, DS327A, DS328A  DT08* | DNPR |
|  |  | ICD-8 | 80511-80599,  80621-80699 | DNPR |
|  | Upper arm | ICD-10 | DS422*, DS423* | DNPR |
|  |  | ICD-8 | 81200-81219 | DNPR |
|  | Forearm | ICD-10 | DS525*, DS526 | DNPR |
|  |  | ICD-8 | 81320-81399 | DNPR |

*DNPR, Danish National Patient Registry; *Including substrings.*

**Codebook 2 - Event definition and algorithm for subsequent fracture ascertainment**

| **Label** | **Anatomical location** | **Definition** | **Data source** |
| --- | --- | --- | --- |
| Pre-transplant fracture /  Prevalent fracture | Any | One or more hospital contacts with a fracture diagnosis (as specified in Codebook 1) up to 10 years before the date of transplantation. | DNPR |
| Incident fracture | Any | Any first contact with a fracture diagnosis after transplantation. The date of this first contact will be noted as the date of fracture.  A contact with a fracture diagnosis following this date is counted as the same episode if the timespan is < 90 days (same anatomical location) or < 30 days (different anatomical location). In the case of a contact meeting one of these criteria, the time window is extended and restarted from the date of this contact. The same criteria are then applied for categorizing any next contact with a fracture diagnosis, and evaluation of contacts continues in this manner^1^. | DNPR |
| Subsequent fracture | Same anatomical location | Next contact with a fracture diagnosis after a fracture-free window of 90 days following the last contact date of the last fracture episode. | DNPR |
|  | Different anatomical location | Next contact with a fracture diagnosis after a fracture-free window of 30 days following the last contact date of the last fracture episode. | DNPR |

*DNPR, Danish National Patient Registry;*

**Codebook 3 – Covariates**

The codes listed were used to identify primary kidney disease as registered in the DNR and comorbidity as registered in the DNPR up to 10 years before date of transplantation. DXA scan registrations were considered up to 1 year before and 1 year after the date of transplantation. For follow-up, DXA scan registrations were considered from date of first post-transplant fracture up to 1 year later.

| **Variable** | **Subgroups** | **Type of code** | **Code list** | **Data source** |
| --- | --- | --- | --- | --- |
| Primary kidney disease/diagnosis | Glomerular disease | PRD (ERA, Registry, 2018^2^) | 1003, 1472, 1464, 1440, 1438, 1088, 1057, 1042, 1035, 1061, 1026, 1570, 1562, 1591, 1589, 1074, 1331, 1558, 1543, 1308, 1298, 1280, 1279, 1144, 1137, 1354, 3754, 3765, 1320, 1312, 3777, 1377, 3749, 1365, 1417, 1401, 1515, 1504, 1251, 1128, 1116, 1163, 1159, 1171, 1205, 1185, 1214, 1192, 1349, 1222, 1233, 1246, 1429, 1100, 1090, 3615, 3604, 1019, 1455, 1267, 1536, 1527, 1493, 1486, 1383, 3847, 3852, 1396 | DNR |
|  | Tubolointerstitial disease | PRD | 1768, 1752, 2196, 2183, 1995, 1982, 1710, 1845, 1832, 2203, 3636, 1625, 1706, 1660, 1673, 2005, 2014, 1850, 1911, 1907, 1618, 1656, 2257, 3662, 1813, 2177, 2165, 2242, 1866, 1723, 1639, 2098, 2080, 2112, 2108, 2033, 2022, 2051, 2046, 2131, 2120, 2154, 2149, 2079, 2067, 2288, 2274, 1799, 1809, 1781, 1775, 1734, 2300, 1687, 1602, 2219, 2226, 3627, 1641, 1747, 3796, 3783, 3806, 1976, 1969, 2235, 3689, 1821, 3670, 2290, 1694, 1897, 1884, 1930, 1924, 1953, 1948, 1878, 2261 | DNR |
|  | Diabetes Mellitus | PRD | 2328, 2316, 2344, 2337 | DNR |
|  | Renal vascular disease /Hypertension | PRD | 2392, 2448, 2430, 2363, 2359, 2453, 2407, 2411, 2385, 2371, 2469, 2424 | DNR |
|  | Other systemic disease | PRD | 2513, 2521, 2623, 2482, 2652, 2566, 2550, 2545, 2532, 2668, 2675, 2610, 2647, 2495, 2606, 2597, 2584, 2578, 2681, 3834, 2509, 2476, 2702, 2699, 2634 | DNR |
|  | Familial /Hereditary Nephropathies | PRD | 3071, 2760, 2756, 3118, 2718, 2725, 2739, 2741, 3085, 2773, 3322, 2794, 2964, 2955, 2929, 3028, 3037, 3230, 3224, 3173, 3160, 3187, 3379, 3314, 3092, 3125, 3305, 2993, 2986, 3139, 2940, 2972, 3810, 3351, 3059, 3102, 2938, 2804, 2815, 3295, 3367, 3253, 3044, 2836, 2843, 2858, 2862, 2870, 2889, 2891, 3063, 2901, 3194, 3207, 3211, 3731, 3000, 3016, 3141, 3156, 3658, 3269, 2787, 3346, 3276, 2917, 2827, 3282, 3333, 3248 | DNR |
|  | Miscellanous Reanl Disorder (categorized as unspecified) | PRD | 3442, 3380, 3403, 3398, 3435, 3426, 3419, 3457, 3564, 3555, 3529, 3540, 3538, 3708, 3643, 3572, 3823, 3712, 3720, 3461, 3501, 3474, 3691, 3517, 3488, 3490 | DNR |
| Cardiovascular disease | Atrial fibrillation or flutter | ICD-10 | I48* | DNPR |
|  |  | ICD-8 | 42793, 42794 | DNPR |
|  | Ischemic heart  disease | ICD-10 | I20*, I21*, I22*, I23*, I251, I259 | DNPR |
|  |  | ICD-8 | 410, 411,413 | DNPR |
|  | Heart failure | ICD-10 | I50*, I110, I130, I132, I420,  I426, I427, I428, I429 | DNPR |
|  |  | ICD-8 | 42709, 42710, 42711,  42719, 42899, 78249 | DNPR |
|  | Stroke | ICD-10 | I60*, I61*, I63*, I64* | DNPR |
|  |  | ICD-8 | 430, 431, 433,434 | DNPR |
|  | Peripheral artery  disease | ICD-10 | I70*, I71*, I72*, I73*, I74*, I77* | DNPR |
|  |  | ICD-8 | 440, 441, 442, 443, 444,  445 | DNPR |
| Hypertension |  | ICD-10 | I10*, I11*, I12*, I13*, I14*, I15* | DNPR |
|  |  | ICD-8 | 400, 401, 402, 403, 404 | DNPR |
| Diabetes |  | ICD-10 | E10*, E11*, E12*, E13*, E14*,  O240, O241, O242, O243,  O245, O249, G632, H360,  N083 | DNPR |
|  |  | ICD-8 | 24900, 24906, 24907,  24909, 25000, 25006,  25007, 25009 | DNPR |
|  |  | ATC | A10A*, A10B-H, A10BX | LSR |
|  | Diabetes type 1 | ICD-8, ICD.10, ATC | Diagnosis of any diabetes as defined above AND ATC-codes are only A10A* (insulin) AND any registration before age 28. | DNPR/LSR |
|  | Diabetes type 2 | ICD-8, ICD.10, ATC | Diagnosis of any diabetes as defined above AND not meeting criteria of “diabetes type 1”. | DNPR/LSR |
| Surgical parathyroidectomy |  | NSCP | KBBA40,  KBBA42,  KBBA50,  KBBA70 | DNPR |
|  |  | Surgery codes (before 1996) | 08440  08450  08460  08510  08520  08560 | DNPR |
| DXA scan, performed |  | Procedure code | UXRE80,  UXRE82,  UXRE85,  UXRG80  UXRF80 | DNPR |

*DNR, Danish Nephrology Registry; DNPR, Danish National Patient Registry; DXA, Dual-energy X-ray Absorptiometry; PRD, Primary Renal Diagnosis; ERA, European Renal Association; LSR, Danish National Prescription Registry; ATC code, Anatomical Therapeutic Chemical code; *Including substrings.*

**Codebook 4 – Anti-osteoporosis medication**

Medical treatment as registered in the Danish National Prescription Registry (LSR) or by in-hospital codes in the Danish National Patient Registry (DNPR). A registration of either code was considered up to 1 year before and 1 year after the date of transplantation. For follow-up, registrations were considered from date of first post-transplant fracture up to 1 year later.

| **Variable** | **Subgroups** | **Type of code** | **Code list** | **Data source** |
| --- | --- | --- | --- | --- |
| Anti-osteoporosis treatment | Bisphosphonates, | ATC | M05BA*, M05BB* | LSR |
|  |  | SKS/Procedure Code | BWHB40* | DNPR |
|  | Denosumab, | ATC-code | M05BX04 | LSR |
|  |  | SKS/Procedure Code | BWHB42 | DNPR |
|  | Romosozumab | ATC | M05BX06 | LSR |
|  | Strontium ranelate | ATC | M05BX03 | LSR |
|  | Teriparatide | ATC | H05AA02 | LSR |

*ATC code, Anatomical Therapeutic Chemical code; SKS code, Sundhedsvæsenets Klassifikationssystem; DNPR, Danish National Patient Registry; LSR, Danish National Prescription Registry; *Including substrings.*

## 2 Supplemental tables

### Suppl table S1. Immunosuppressive treatment protocols for patients of standard immunological risk by transplant center.

| **Center** | **Induction** | **Maintenance** |
| --- | --- | --- |
| **Herlev, Copenhagen**^3^ |  |  |
| 2000-2004 | 2000 ATGAM  2001, June, Thymoglobulin  Methylprednisolone | Ciclosporin  Azathioprine  Prednisolone |
| 2004-2010 | 2004, April, Daclizumab  2008, November, Basiliximab  Methylprednisolone | Ciclosporin  MMF  Prednisolone |
| **Rigshospitalet, Copenhagen** |  |  |
| 2000-2010 | 2000 ATGAM  2001, June, Thymoglobulin  2004, April, Daclizumab  2008, November, Basiliximab  Methylprednisolone | Ciclosporin  MMF  Prednisolone |
| 2011, March – present | Basiliximab  Methylprednisolone | Tac  MMF  Prednisolone |
| **Odense** ^4,5^ |  |  |
| 2000-2010 | Basiliximab | Ciclosporin  MMF (2000mg/day) |
| 2011- present | Basiliximab | Tac (target 5ug/L)  MMF 1500mg/day |
| Patient group: IgA-nephropathy (period unspecified) | Thymoglobulin  Methylprednisolone | Tac (target 5ug/L)  MMF 1500mg/day |
| **Aarhus^6^** |  |  |
| 2000 – 2004 | Basiliximab  Methylprednisolone | Tac (target 3-5ug/L)  MMF  Prednisolone |
| 2004 – present | Basiliximab  Methylprednisolone | Tac (target 3-5ug/L)  MMF 1000mg/day  Prednisolone |

*ATGAM, lymphocyte immune globulin, anti-thymocyte globulin; MMF, Mycophenolate mofetil; Tac, Tacrolimus;*

### Suppl table S2. Baseline characteristics. The population is stratified by post-transplant fracture event.

|  | Baseline characteristics of Danish kidney transplant recipients by post-transplant fracture event | | | |
| --- | --- | --- | --- | --- |
|  | | Total | Fracture^1^ | No fracture^1^ |
| **Number of patients** | | 3,977 | 788 | 3,189 |
| **Age, years** | | 50 (40, 60) | 52 (41, 61) | 50 (39, 60) |
| **Age groups** | |  |  |  |
| 18-39 | | 990 (24.9%) | 169 (21.4%) | 821 (25.7%) |
| 40-59 | | 1,947 (49.0%) | 382 (48.5%) | 1,565 (49.1%) |
| >60 | | 1,040 (26.2%) | 237 (30.1%) | 803 (25.2%) |
| **Sex, female** | | 1,487 (37.4%) | 347 (44.0%) | 1,140 (35.7%) |
| **Calendar year of transplantation** | |  |  |  |
| 2000-2004 | | 672 (16.9%) | 214 (27.2%) | 458 (14.4%) |
| 2005-2010 | | 905 (22.8%) | 244 (31.0%) | 661 (20.7%) |
| 2011-2016 | | 1,175 (29.5%) | 247 (31.3%) | 928 (29.1%) |
| 2017-2022 | | 1,225 (30.8%) | 83 (10.5%) | 1,142 (35.8%) |
| **Transplant Center** | |  |  |  |
| Herlev Hospital, Copenhagen | | 193 (4.9%) | 60 (7.6%) | 133 (4.2%) |
| Odense University Hospital | | 1,006 (25.3%) | 167 (21.2%) | 839 (26.3%) |
| Rigshospitalet, Copenhagen | | 1,344 (33.8%) | 268 (34.0%) | 1,076 (33.7%) |
| Aarhus University Hospital | | 1,434 (36.1%) | 293 (37.2%) | 1,141 (35.8%) |
| **Primary kidney disease** | |  |  |  |
| Diabetes Mellitus | | 586 (14.7%) | 161 (20.4%) | 425 (13.3%) |
| Familial / Hereditary nephropathies | | 695 (17.5%) | 150 (19.0%) | 545 (17.1%) |
| Glomerular disease | | 1,164 (29.3%) | 189 (24.0%) | 975 (30.6%) |
| Renal vascular disease / Hypertension | | 400 (10.1%) | 67 (8.5%) | 333 (10.4%) |
| Tubulointerstitial disease | | 334 (8.4%) | 65 (8.2%) | 269 (8.4%) |
| Unspecified and systemic disease collapsed | | 798(20.1%) | 156 (19.8%) | 642 (20.1%) |
| **Pretransplant Dialysis Therapy** | |  |  |  |
| Hemodialysis | | 1,957 (49.2%) | 405 (51.4%) | 1,552 (48.7%) |
| Peritoneal dialysis | | 1,227 (30.9%) | 259 (32.9%) | 968 (30.4%) |
| Pre-emptive | | 793 (19.9%) | 124 (15.7%) | 669 (21.0%) |
| **Time on dialysis** | |  |  |  |
| Short dialysis therapy | | 1,346 (33.8%) | 232 (29.4%) | 1,114 (34.9%) |
| Long dialysis therapy | | 1,838 (46.2%) | 432 (54.8%) | 1,406 (44.1%) |
| Pre-emptive | | 793 (19.9%) | 124 (15.7%) | 669 (21.0%) |
| **Dialysis vintage, months** | | 22 (11, 42) | 26 (13, 45) | 21 (10, 42) |
| **Donor type** | |  |  |  |
| Deceased donor | | 2,591 (65.1%) | 563 (71.4%) | 2,028 (63.6%) |
| Living donor | | 1,386 (34.9%) | 225 (28.6%) | 1,161 (36.4%) |
| **Cold ischemic time, hours** | | 11.5 (3.9, 17.3) | 13.4 (4.5, 18.3) | 11.0 (3.9, 17.0) |
| **Charlson Comorbidity Index** | | 2.0 (2.0, 4.0) | 3.0 (2.0, 4.0) | 2.0 (2.0, 4.0) |
| **History of hypertension** | | 2,809 (70.6%) | 568 (72.1%) | 2,241 (70.3%) |
| **History of cardiovascular disease** | | 1,405 (35.3%) | 304 (38.6%) | 1,101 (34.5%) |
| **History of diabetes** | | 901 (22.7%) | 234 (29.7%) | 667 (20.9%) |
| **Diabetes types** | |  |  |  |
| No diabetes | | 3,076 (77.3%) | 554 (70.3%) | 2,522 (79.1%) |
| Type 1 | | 308 (7.7%) | 105 (13.3%) | 203 (6.4%) |
| Type 2 | | 593 (14.9%) | 129 (16.4%) | 464 (14.6%) |
| **History of any fracture** | | 503 (12.6%) | 150 (19.0%) | 353 (11.1%) |
| **History of MOF** | | 176 (4.4%) | 63 (8.0%) | 113 (3.5%) |
| **Number of fracture events** | |  |  |  |
| =>2 | | 92 (18.3%) | 35 (23.3%) | 57 (16.1%) |
| 1 | | 411 (81.7%) | 115 (76.7%) | 296 (83.9%) |
| **Time from last fracture to transplantation, years** | | 4.0 (1.8, 6.7) | 3.4 (1.8, 6.7) | 4.3 (1.8, 6.7) |
| **History of parathyroidectomy** | | 222 (5.6%) | 41 (5.2%) | 181 (5.7%) |
| **DXA around time of transplantation** | | 633 (15.9%) | 135 (17.1%) | 498 (15.6%) |
| **Anti-osteoporosis medication around time of transplantation** | | 54 (1.4%) | 18 (2.3%) | 36 (1.1%) |
| *^1^Continous variables are presented as median (IQR). Categorical variables are presented as n (%).*  *Short and long dialysis therapy defined by a 18-month cut-off.* | | | | |

### Suppl table S3. 5- and 10-year risks of any fracture and MOF by transplant center, patients transplanted at Herlev excluded.

| **Risk of any fracture** |  |  |
| --- | --- | --- |
|  | 5-year cumulative incidence,  % (95% CI) | 10-year cumulative incidence, % (95% CI) |
| Odense (n = 1,006) | 11.1% (9.1%, 13.3%) | 18.7% (15.9%, 21.6%) |
| Rigshospitalet, Aarhus (n = 2,778) | 13.3% (12.0%, 14.7%) | 22.3% (20.5%, 24.2%) |
| **Risk of MOF** | | |
|  | 5-year cumulative incidence, % (95% CI) | 10-year cumulative incidence, % (95% CI) |
| Odense (n = 1,006) | 4.2% (3.0%, 5.7%) | 8.3% (6.4%, 10.5%) |
| Rigshospitalet, Aarhus (n = 2,778) | 5.3% (4.5%, 6.3%) | 9.5% (8.2%, 10.8%) |

### Suppl table S4. Baseline characteristics by categories of transplant centers, patients transplanted at Herlev excluded.

| Baseline characteristics of Danish kidney transplant recipients by transplant center (Odense versus Rigshospitalet and Aarhus) | | |
| --- | --- | --- |
|  | Odense^1^ | Rigshospitalet and Aarhus^1^ |
| **Number of patients** | 1,006 | 2,778 |
| **Age, years** | 51 (40, 60) | 50 (39, 60) |
| **Age groups** |  |  |
| 18-39 | 228 (22.7%) | 719 (25.9%) |
| 40-59 | 506 (50.3%) | 1,340 (48.2%) |
| >60 | 272 (27.0%) | 719 (25.9%) |
| **Sex, female** | 367 (36.5%) | 1,041 (37.5%) |
| **Calendar year of transplantation** |  |  |
| 2000-2004 | 132 (13.1%) | 436 (15.7%) |
| 2005-2010 | 195 (19.4%) | 621 (22.4%) |
| 2011-2016 | 332 (33.0%) | 843 (30.3%) |
| 2017-2022 | 347 (34.5%) | 878 (31.6%) |
| **Primary kidney disease** |  |  |
| Diabetes Mellitus | 157 (15.6%) | 387 (13.9%) |
| Familial / Hereditary nephropathies | 173 (17.2%) | 493 (17.7%) |
| Glomerular disease | 304 (30.2%) | 814 (29.3%) |
| Renal vascular disease / Hypertension | 93 (9.2%) | 285 (10.3%) |
| Tubulointerstitial disease | 80 (8.0%) | 240 (8.6%) |
| Unspecified and systemic disease collapsed | 199 (19.8%) | 559 (20.1%) |
| **Pretransplant Dialysis Therapy** |  |  |
| Hemodialysis | 501 (49.8%) | 1,383 (49.8%) |
| Peritoneal dialysis | 312 (31.0%) | 839 (30.2%) |
| Pre-emptive | 193 (19.2%) | 556 (20.0%) |
| **Time on dialysis** |  |  |
| Short dialysis therapy | 375 (37.3%) | 898 (32.3%) |
| Long dialysis therapy | 438 (43.5%) | 1,324 (47.7%) |
| Pre-emptive | 193 (19.2%) | 556 (20.0%) |
| **Dialysis vintage, months** | 20 (10, 39) | 23 (11, 44) |
| **Donor type** |  |  |
| Deceased donor | 583 (58.0%) | 1,877 (67.6%) |
| Living donor | 423 (42.0%) | 901 (32.4%) |
| **Cold ischemic time, hours** | 13.6 (9.8, 17.0) | 11.0 (3.7, 17.4) |
| **Charlson Comorbidity Index** | 3.0 (2.0, 4.0) | 2.0 (2.0, 4.0) |
| **History of hypertension** | 720 (71.6%) | 1,969 (70.9%) |
| **History of cardiovascular disease** | 351 (34.9%) | 977 (35.2%) |
| **History of diabetes** | 245 (24.4%) | 607 (21.9%) |
| **Diabetes types** |  |  |
| No diabetes | 761 (75.6%) | 2,171 (78.1%) |
| Type 1 | 82 (8.2%) | 198 (7.1%) |
| Type 2 | 163 (16.2%) | 409 (14.7%) |
| **History of any fracture** | 133 (13.2%) | 338 (12.2%) |
| **History of MOF** | 47 (4.7%) | 118 (4.2%) |
| **Time from last fracture to transplantation, years** | 3.4 (1.5, 6.8) | 4.2 (1.8, 6.8) |
| **History of parathyroidectomy** | 37 (3.7%) | 174 (6.3%) |
| **DXA around time of transplantation** | 193 (19.2%) | 361 (13.0%) |
| **Anti-osteoporosis medication around time of transplantation** | 6 (0.6%) | 46 (1.7%) |
| ^1^Continuous variables are presented as median (IQR). Categorical variables are presented as n (%) | | |
|  | | |

### Suppl table S5. Risk of subsequent fracture by fracture location

| Risk of any subsequent fracture by location of first fracture | N at risk | 2-year cumulative  incidence, (95% CI) |
| --- | --- | --- |
| **Location of first fracture** |  |  |
| Lower leg and knee | 144 | 14% (8.6%, 20%) |
| Forearm | 111 | 9.0% (4.4%, 16%) |
| Foot | 99 | 15% (8.2%, 23%) |
| Hand | 93 | 14% (7.4%, 22%) |
| Multiple | 66 | 13% (6.0%, 23%) |
| Hip | 63 | 17% (8.7%, 28%) |
| Upper arm | 61 | 12% (5.3%, 22%) |
| Other arm | 44 | 9.4% (2.9%, 21%) |
| Thoracolumbar spine | 26 | 20% (7.0%, 38%) |
| Shoulder and clavicula | 26 | 8.2% (1.3%, 23%) |
| Ribs, sternum and cervical spine | 23 | 4.8% (0.3%, 21%) |
| Pelvis | 22 | 20% (5.9%, 41%) |
| Other femur (not hip) | 10 | 16% (0.44%, 53%) |

### Suppl table S6. Sensitivity analysis: Follow-up ending when graft loss

|  | 5-year cumulative  incidence, % (95% CI) | 10-year cumulative  incidence, % (95% CI) | Incidence rate per  1000 py (95% CI) |
| --- | --- | --- | --- |
|  |  |  |  |
| Any fracture (n =3997) | 11.8 % (95% CI 10.8-12.9) | 18.7% (95% CI 17.4-20.2) | 25.6 (95% CI 23.7-27.6) |
| MOF (n=3997 | 4.7 % (95% CI 4.0-5.4) | 8.2% (95% CI 7.2-9.2) | 10.9 (95% CI 9.4-11.9) |

## 3 Supplemental Figures

### Suppl. Figure S1. Cumulative incidence of any first post-transplant fracture.


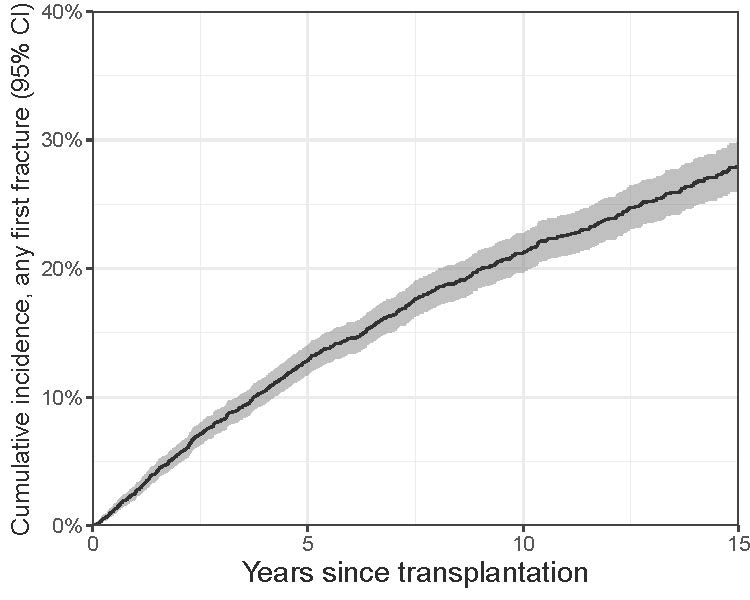


### Suppl. Figure S2. Incidence rates for any first post-transplant fracture by 6-month intervals following transplantation date.


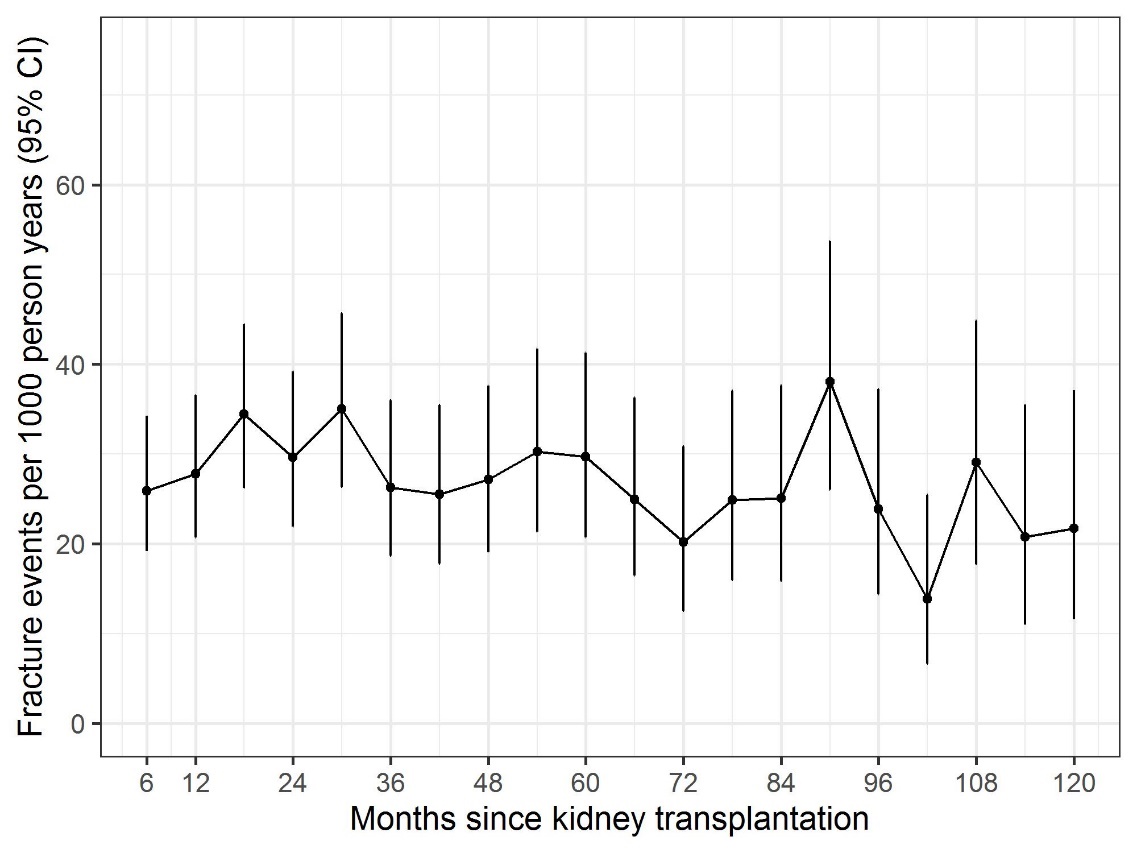


### Suppl. Figure S3. Crude and standardized incidence rates for first major osteoporotic fracture by calendar period of kidney transplantation. Estimates restricted to 5 years of follow-up.


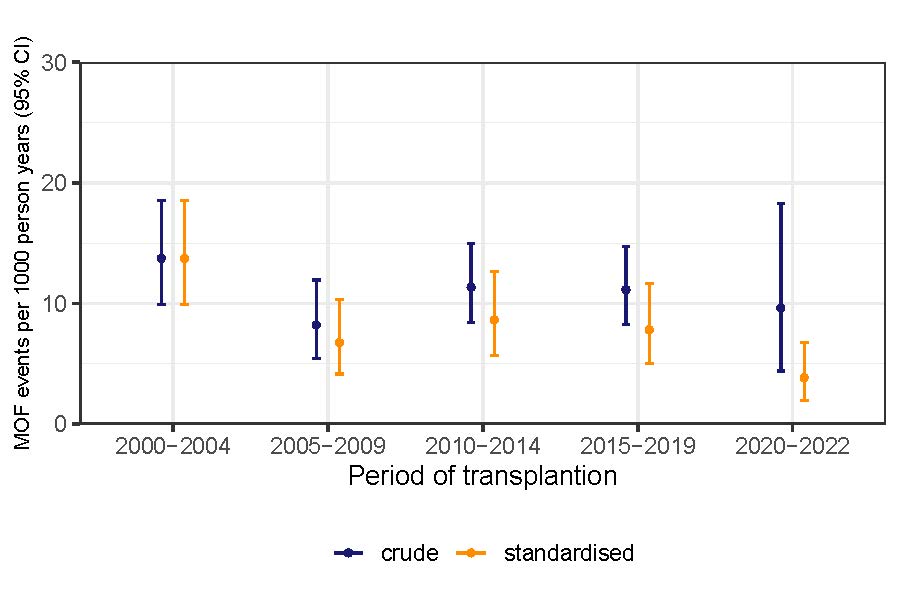


### Suppl. Figure S4. Cumulative incidence of any first post-transplant fracture by early rejection status.


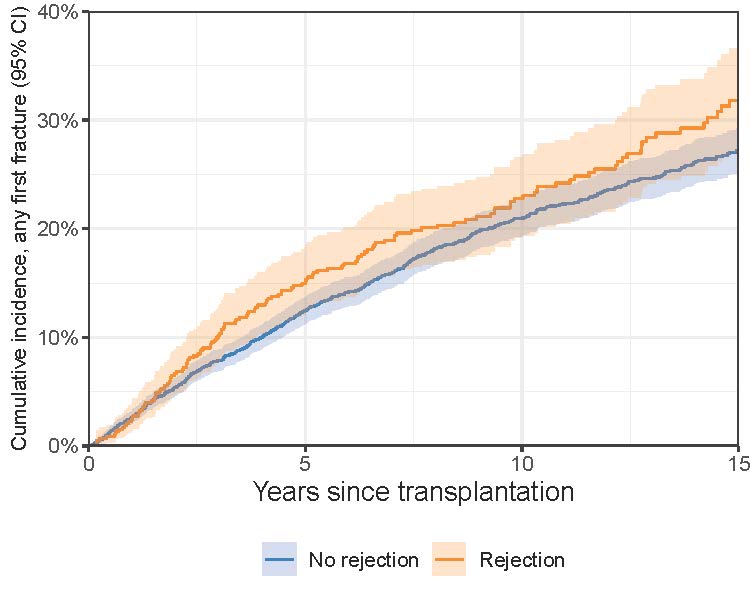


### Suppl. Figure S5. Cumulative incidence of any first post-transplant fracture by transplant center


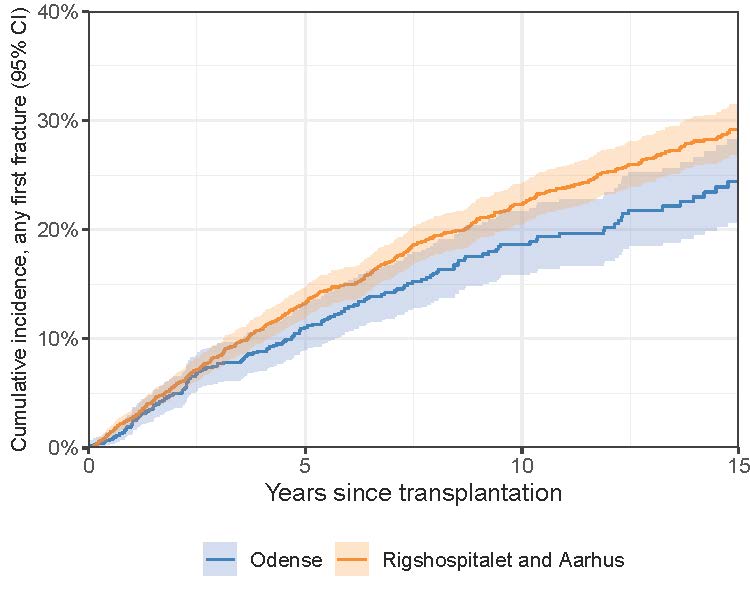


### Suppl. Figure S6. Incidence rates for any subsequent fracture following a first post-transplant fracture by 6-month intervals after date of first fracture event.


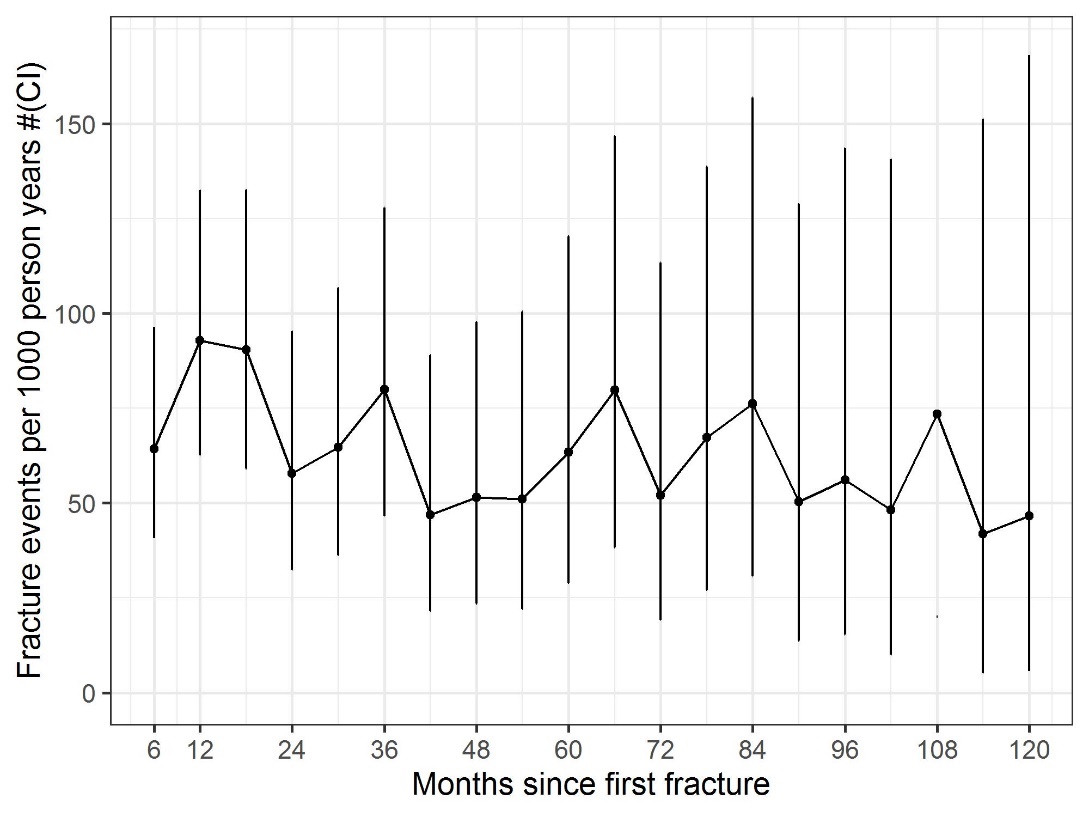


## 4. References

1 Wright, N. C. *et al.* The Design and Validation of a New Algorithm to Identify Incident Fractures in Administrative Claims Data. *J Bone Miner Res* **34**, 1798-1807, doi:10.1002/jbmr.3807 (2019).

2 Tomson, C. R. V. *ERA Registry: 2018 Primary Renal Disease (PRD) codes*, 2018).

3 Frederiksen, A. M., Hansen, J. M., Ewers, B., Gasbjerg, A. & Marckmann, P. Enkeltcenteropgørelse af nyretransplanterede patienters nyrefunktion og immunsuppressive behandling. *Ugeskrift for Laeger* **170/19** (2008).

4 El-Faramawi, M., Rohr, N. & Jespersen, B. Steroid-free immunosuppression after renal transplantation-long-term experience from a single centre. *Nephrol Dial Transplant* **21**, 1966-1973, doi:10.1093/ndt/gfl131 (2006).

5 Saleh, Q. W., Gronbaek, L., Kronborg, C., Lauridsen, J. T. & Tepel, M. Kidney function, future health costs, and quality-adjusted life-years in kidney transplant recipients transplanted during the SARS-Cov-2 lockdown in Denmark - An observational study. *Heliyon* **7**, e08489, doi:10.1016/j.heliyon.2021.e08489 (2021).

6 Ekberg, J. *et al.* A Randomized Controlled Trial on Safety of Steroid Avoidance in Immunologically Low-Risk Kidney Transplant Recipients. *Kidney Int Rep* **7**, 259-269, doi:10.1016/j.ekir.2021.11.028 (2022).
